# Knowledge of iatrogenic premature ovarian insufficiency among Chinese obstetricians and gynaecologists: a national questionnaire survey

**DOI:** 10.1186/s13048-020-00739-z

**Published:** 2020-11-18

**Authors:** Yanfang Wang, Ying Zou, Wei Wang, Qingmei Zheng, Ying Feng, Han Dong, Zhangyun Tan, Xiaoqin Zeng, Yinqing Zhao, Danhong Peng, Xiaomin Yang, Aijun Sun

**Affiliations:** 1 Department of Obstetrics and Gynaecology, Peking Union Medical College Hospital (East), Chinese Academy of Medical Sciences and Peking Union Medical College, No. 1 Shuaifuyuan, Dongcheng District, Beijing, 100730 China; 2 Hunan Provincial Maternal and Child Health Care Hospital, Changsha, 410008 Hunan China; 3 The Second Hospital of Hebei Medical University, Shijiazhuang, 050000 Hebei China; 4 The Affiliated Hospital of Qingdao University, Qingdao, 266500 Shandong China; 5 The Second Affiliated Hospital of Nanchang University, Nanchang, 330006 Jiangxi China; 6 Women and Children’s Hospital of Jinzhou, Jinzhou, 121000 Liaoning China; 7 Xinhui Maternity and Children’s Hospital, Nanning, 529100 Guangxi China; 8 Guangzhou Women and Children’s Medical Center, Guangzhou, 510000 Guangdong China; 9 Zhongda Hospital Southeast University, Nanjing, 210009 Jiangsu China; 10 Liuzhou Maternity and Child Healthcare Hospital, Liuzhou, 545001 Guangxi China

**Keywords:** Premature ovarian insufficiency, Iatrogenic menopause, Ovarian impairment, Knowledge, National survey

## Abstract

**Background:**

With increasing cases of iatrogenic premature ovarian insufficiency (POI), more clinicians are required to counsel patients regarding the gonadotoxic effects of iatrogenic treatments. This survey aimed to explore obstetricians and gynaecologists’ knowledge regarding iatrogenic POI. A national online questionnaire survey was conducted across China. Respondents were asked to select the iatrogenic condition(s) that can cause POI based on their experience and knowledge.

**Results:**

Of the 5523 returned questionnaires, 4995 were analysed. Among tumour therapies causing POI, most respondents agreed that radiotherapy (73.5% of respondents) and chemotherapy (64.1%) are risk factors for POI. While only 6.5 and 7.8% of the gynaecological oncologists believed that tumour immunotherapy and tumour-targeting therapy, respectively, may cause ovarian impairment, 31.8 and 22.2% of the non-gynaecologic oncologists believed that these therapies could affect ovarian health. Most respondents believed that ovarian cystectomy (54.4%) was a risk factor for POI. In contrast, only a few respondents believed that hysterectomy with bilateral salpingectomy (39.6%) and uterine artery embolisation (33.5%) could cause ovarian impairment. Only 30.5% of respondents believed that immunosuppressants (ISs) increased the risk of POI. Views differed with experience and hospital setting.

**Conclusions:**

The knowledge of gonadal toxicity due to traditional tumour treatments is generally high among Chinese obstetricians and gynaecologists. A misunderstanding may exist in primary care hospitals and general gynaecologists regarding a link between novel tumour treatments and POI, owing to the lack of convincing evidence. Knowledge of POI caused by hysterectomy and ISs should be improved.

**Supplementary Information:**

The online version contains supplementary material available at 10.1186/s13048-020-00739-z.

## Background

Premature ovarian insufficiency (POI) is a clinical syndrome defined by loss of ovarian activity before the age of 40 years [1]. The incidence of spontaneous POI, typically assumed to occur in approximately 1% of adult women [2], has increased to 2.4–2.8% in recent years [3, 4]. As a consequence of being exposed to lower estrogen for a longer period, women with POI have an increased risk of premature morbidity and mortality [5], cardiovascular and cerebrovascular diseases [6, 7], osteoporosis [8, 9], impaired cognition [10], and diminished sexual health [11]. However, the etiologies of POI are largely unknown. Of the few identified causes, iatrogenic conditions account for a large proportion (~ 65%) of the cases [12], including radiotherapy (RT), chemotherapy (CT), and drugs for various autoimmune diseases [13, 14]. The incidence of iatrogenic POI has been reported to be growing owing to increasing survival rates following diverse cancer treatments [15–17]. In addition, accumulating evidence has revealed that women with endometriosis or a history of pelvic surgery are more likely to experience severely compromised ovarian function, including POI [18, 19]. In summary, the increasing prevalence of iatrogenic POI has posed great challenges to clinicians, especially obstetricians and gynaecologists.

Fortunately, iatrogenic POI can be partially avoided or reduced using many preventative measures, including optimisation of CT regimens or radiation field, application of gonadotropin-releasing hormone agonist (GnRH-a), and fertility-sparing surgical strategies, all of which are implemented by physicians. It has been continually emphasised that proper and comprehensive fertility counselling should be provided to patients who are within their reproductive ages prior to starting iatrogenic treatment to inform them on both the risk of treatment-related gonadotoxicity and the potential future needs of accessing an assisted reproductive clinic. Therefore, oncofertility counselling of concerned patients has already been included in international guidelines including but not limited to the American Society of Clinical Oncology (ASCO) Clinical Practice Guideline [20] and the European Society of Medical Oncology (ESMO) Clinical Practice Guideline [21]. To this end, the knowledge of iatrogenic POI among health care providers/clinicians is particularly important. Therefore, we surveyed obstetricians and gynaecologists across China about their general knowledge of iatrogenic POI to fully investigate the current understanding of this condition and any underlying challenges in this population.

## Results

### General background information of respondents

Of the 5524 questionnaires that were returned, 249 were answered by respondents who were neither obstetricians nor gynaecologists and were excluded, leaving a qualified sample of 4995 (95.5%). General background information about the respondents is presented in Table 1. Most respondents were women (96.2%), aged 36–55 years (69.2%), with over 10 years of working experience. Regarding respondents’ work setting, 34.9 and 49.0% of respondents worked in tertiary and secondary hospitals, respectively. Meanwhile, 51.5% of respondents practice at general hospitals, and 42.9% practice at maternity and children hospitals or reproductive hospitals. Most participants (79.5%) specialised in gynaecology, including reproductive endocrinologists, obstetricians-gynaecologists (who work as a gynaecologist and an obstetrician simultaneously), and gynaecologists.

**Table 1:** General background information about the respondents

Category	N (%)
Gender	
Female	4805 (96.2)
Male	190 (3.8)
Age in years	
18–25	112 (2.2)
26–35	1079 (21.6)
36–45	2065 (41.3)
46–55	1529 (30.6)
> 55	210 (4.2)
Length of service (years)	
≤ 5	677 (13.6)
6 ~ 10	861 (17.2)
11 ~ 20	1517 (30.4)
> 20	1940 (38.8)
Hospital level	
Tertiary hospital	1745 (34.9)
Secondary hospital	2445 (49.0)
Community hospital or others	448 (16.1)
Hospital type	
General hospital	2572 (51.5)
Maternity and child care hospital	2144 (42.9)
Others	279 (5.6)
Speciality type	
General gynaecologist	3400 (68.1)
Gynaecologic or reproductive endocrinologist	345 (6.9)
Obstetrician-gynaecologist	147 (2.9)
Gynaecologic oncologist	77 (1.54)
Obstetrician	1026 (20.5)
Total	4995 (100)

### Iatrogenic condition(s) to induce POI

A summary of Chinese obstetricians and gynaecologists’ views on iatrogenic condition(s) that can induce POI is presented in Table 2. Among the tumour therapies respondents thought would induce POI, RT ranked first (73.5%), followed by CT (64.1%), tumour immunotherapy (TIT) (31.8%), and tumour-targeting therapy (TTT; 22.2%) (*p* < 0.05).

**Table 2:** Chinese obstetricians and gynaecologists’ views on iatrogenic condition(s) to induce POI

Variable	N (%)	χ^2^	p
**Tumour therapies**			
Radiotherapy	3669 (73.5)	1459.1	< 0.01
Chemotherapy	3202 (64.1)		
Tumour immunotherapy	1590 (31.8)		
Tumour-targeting therapy	1109 (22.2)		
**Surgeries or procedures**			
Ovarian cystectomy	2716 (54.4)	1288.4	< 0.01
Hysterectomy with bilateral salpingectomy	1980 (39.6)		
Uterine artery embolisation	1675 (33.5)		
Bilateral salpingectomy	1246 (24.9)		
Bilateral tubal ligation	779 (15.6)		
**Others**			
Immunosuppressants	1523 (30.5)	/	/

Most respondents believed that ovarian cystectomy (OC) could have an adverse impact on ovarian reserve (54.4% of respondents). In contrast, a minority believed that hysterectomy with bilateral salpingectomy (H&BS; 39.6%), uterine artery embolisation (UAE; 33.5%), bilateral salpingectomy (BS; 24.9%), or bilateral tubal ligation (BTL; 15.6%, *P* < 0.05) could have an adverse impact on ovarian reserve. Only 30.5% respondents believed that immunosuppressants (ISs) could lead to an increased risk of POI.

### Influencing factors

**Fig. 1:**
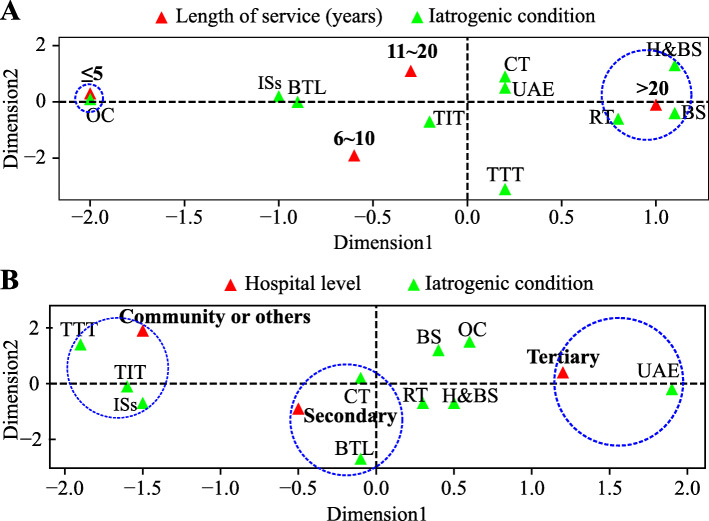
Visualisation of the correspondence analysis by physician’s length of service (**a**), and level of hospital (**b**). The interpretation rate in each dimension is: 87.7% (Dimension 1) and 7.50% (Dimension 2) in A; and 98.9% (Dimension 1), 1.1% (Dimension 2) in B. More details are available in Supplementary Table 1 and Supplementary Table 2. Abbreviations: RT: radiotherapy; CT: chemotherapy; TIT: tumour immunotherapy; TTT: tumour-targeting therapy; OC: ovarian cystectomy; H&BS: hysterectomy with bilateral salpingectomy; UAE: uterine artery embolisation; BS: bilateral salpingectomy; BTL: bilateral tubal ligation; ISs: immunosuppressants

The analysis demonstrated that the level of understanding about the risk of POI with different treatments varied with the length of service and hospital setting. A visualisation of the correspondence analysis is shown in Fig. 1. For example, respondents with over 20 years of service were more likely to consider RT, BS, and H&BS as risk factors for POI than those in other age groups. Meanwhile, those with less than 5 years of service typically only indicated the risk of POI caused by OC. Physicians from tertiary hospitals might pay more attention to ovarian impairment caused by UAE, whereas those from secondary hospitals were more aware of the association between ovarian impairment and CT and BTL. Interestingly, TTT, TIT, and IS were considered more often to be risk factors for POI by physicians from community hospital or others, where advanced treatments are less likely to be available. Finally, while 6.5 and 7.8% of the gynaecological oncologists believed that TIT and TTT may adversely affect the ovarian reserve, respectively, 31.8 and 22.2% of non-gynaecologic oncologists believed that the same treatments may be risk factors for POI.

## Discussion

### Tumour therapy

In women, primordial germ cells (PGCs) enter meiosis at week 10, progressing to prophase I and remaining at this stage for a long period until ovulation [22]. This process makes PGCs extremely sensitive to the effects of CT and RT. Chemotherapeutic agents might i) have a detrimental effect on the DNA of primordial follicles; ii) induce overactivation, apoptosis, or atresia of follicles; or iii) cause stromal fibrosis as well as vascular damage in ovaries [23, 24]. Whereas RT induces rapid prolonged primordial follicle loss mostly via ionisation and oxidative stress [25, 26], which can be more damaging to ovarian reserve than CT due to its inevitable off-target effects. It has been reported that the dose required to induce permanent ovarian failure would vary from 20.3 Gy at birth to 14.3 Gy at 30 years in humans [26]. Evidence suggests that the incidence of POI in female survivors of childhood and adolescent cancer ranges from 2.1 to 82.2% [27]. A rapidly growing group of cancer survivors requires the availability of more physicians to better counsel patients regarding the gonadotoxic effects of cancer treatment and provide them with appropriate options. However, a recent study in 2014 demonstrated that while 71% of oncologists were aware of the risk of POI following exposure to alkylating agents, only 15% of primary care physicians were aware of this risk [28]. This nationally representative study observed that there is some general knowledge about tumour-treatment-related POI among Chinese obstetricians and gynaecologists. Among respondents, 73.5 and 64.1% were aware that RT and CT, respectively, are the two main factors leading to the decline of ovarian reserve. Furthermore, respondents with more than 10 years of experience working in secondary or tertiary hospitals had better knowledge regarding risk factors for iatrogenic POI than others. Therefore, relevant fertility-related education should be provided in a more targeted manner in the future.

Recently, novel therapies, including TTT and TIT, have been increasingly used in clinical settings. Despite their high selectivity, they may also affect healthy cells and tissues, including gonadal tissues, as highlighted by animal studies [29]. Unlike the direct toxicity of RT or CT, TTT tends to influence folliculogenesis, PGC establishment, or ovarian follicular growth and differentiation by acting on the corresponding signalling pathways [29]. However, available human data on the association between TTT and ovarian health are limited and heterogeneous [30]. Most existing data are from retrospective evaluations [31] or case reports [32]. For instance, Allegra et al. [31] reported that new-onset POI occurred in 39% of patients treated with a combination of modified FOLFOX-6 and bevacizumab (a type of TTT) compared with 2.6% in the control group treated with modified FOLFOX-6. In this cohort, ovarian function recovered in 86% of patients after cessation of treatment. Regarding TIT, existing data are more limited; however, experts in this field are optimistic about its efficacy [33]. The results of our survey demonstrated that most physicians from community hospitals or others tended to believe that TIT and TTT may cause adverse effects to ovarian reserve, which, however, was not the case for most gynaecological oncologists. These phenomena imply that the popularisation and speed at which relevant information is updated among physicians practising at different level healthcare and specialities vary considerably. With inadequate convincing public data, the chances are slim that primary care physicians will be aware of the risks of novel therapies to ovarian health without continually reviewing the literature. Therefore, to improve the reproductive health and long-term quality of life of cancer patients, both well-designed clinical observations and more accessible information and education for clinicians are required.

### Surgeries or procedures

POI induced by non-oophorectomised surgeries is less common than traditional cancer treatment; however, since these surgeries are regular treatments for many benign gynaecological conditions or early tumours at any age, their impact on fertility should not be ignored. Evidence has confirmed an association between OC and a reduction in ovarian reserve, especially in patients with severe endometriosis [34–36]. Our survey demonstrated that OC-inducing ovarian impairment was commonly recognised among respondents, even in those with less than 5 years of service. This indicates that POI caused by OC may not be uncommon in clinical practice.

Anatomically, H&BS, UAE, BS, and BTL may reduce blood supply to the ovaries to varying degrees, thus having a potential impact on ovarian reserve. Previous studies have demonstrated a certain reduction in ovarian function after H&BS [37, 38]. In 2011, Moorman et al. published their prospective research, including 2410 patients aged between 30 and 47 years, revealing a nearly two-fold increased risk for ovarian failure among women undergoing hysterectomy without bilateral oophorectomy compared with women of similar age with intact uteri (level of evidence: II) [39]. With accelerating menopause and hormone deprivation caused by hysterectomy, an increased overall risk of morbidity and mortality is similarly revealed. A cohort study of 666,588 women demonstrated that hysterectomy without oophorectomy performed before age 35 and H&BS performed before age 45 were associated with an increase in all-cause mortality (hazard ratio, 1.29 and 1.15, respectively) [40] and the incidence of depression [41]. However, in our study, only 39.6% of the obstetricians and gynaecologist respondents were aware of H&BS causing diminished ovarian reserve. From a public health perspective, these are vital issues that require further training and education for health professionals to avoid unnecessary hysterectomy.

Women under age 40 appear unaffected by UAE [42]. Of 7.3% of cases of amenorrhea after UAE, 86% occurred in patients aged 45 years or older [43]. While a meta-analysis of 353 patients demonstrated that UAE may not result in impaired ovarian reserve, regardless of age [44], other studies with small sample sizes demonstrated a contrary result [45, 46]. Our survey demonstrated that 33.5% of respondents were concerned about UAE leading to POI, especially in tertiary hospitals where UAE procedures are completed more frequently. BS and BTL are less likely to induce POI, as they may have no significant short-term effects on ovarian function indicators [47–49]. However, their long-term effect on fertility outcomes remains uncertain [47, 50]. Most respondents remained optimistic about the effects of BS and BTL on ovarian health.

### Immunosuppressants

ISs are often used for long-term treatment of autoimmune diseases, such as systemic lupus erythematosus (SLE), which predominantly affect young women. Some of these agents have distinctive gonadal toxicity. For example, cyclophosphamide has been considered an independent risk factor for POI in SLE patients [13]. During the administration of cyclophosphamide, the incidence of POI was < 50% of women aged below 30 years and 60% of women aged between 30 and 40 years [51]. Similarly, reversible amenorrhea (64.3% of patients) [52] and irregular menstruation (70% of patients) [53] were observed in premenopausal women following exposure to *Tripterygium wilfordii* Hook.f., a well-known Chinese herbal medicine with an immunosuppressive effect. However, 69.5% of respondents to our survey were not aware of the gonadal impairment of IS, which appeared more prevalent among respondents from tertiary hospitals. As IS agents are typically prescribed by an immunologist or an internal medicine physician, we speculate that these differences may be due to a high degree of departmental specialisation in tertiary hospitals. Thus, it is necessary to conduct relevant training and education and strengthen the exchange of experience between different departments on IS use.

## Conclusions

This national survey is the first to explore obstetricians and gynaecologists’ knowledge and awareness regarding iatrogenic POI. Considering the respondents’ background information, the results of this survey not only generate a clearer picture of the understanding of iatrogenic POI in this field but also help us determine the underlying problems in knowledge translation. However, there were some limitations: the proportion of respondents with different backgrounds (gender, speciality, or hospital type) was unevenly distributed; the iatrogenic measures listed in the questionnaire were limited, and we did not give a detailed explanation for the ISs included in the survey. Therefore, further investigations and improved survey design are needed to confirm and update our conclusions. Our survey demonstrated that the knowledge of gonadal toxicity of traditional tumour treatments is generally high among obstetricians and gynaecologists; however, there is still a relative lack of understanding among physicians with less experience and/or from community hospitals. A misunderstanding relating to novel tumour treatments and ovarian health may exist in community hospitals and among non-gynaecologic oncologists due to inadequate convincing evidence. Additionally, the knowledge of POI caused by hysterectomy and ISs needs to be improved. Based on these results, we hope to perform more purposeful and targeted re-training for doctors in the future.

## Methods

### Study setting and implementation

An online survey was administered to obstetricians and gynaecologists across China between June 7 and July 3, 2020. The questionnaire was distributed with the assistance of the China Maternal and Child Health Association, Society of Gynaecological Endocrinology and answered anonymously. The study was reviewed and approved by the Ethics Committee of Peking Union Medical College Hospital (Ethical code number: S-k1189–1; date of approval: May 08, 2020) and is in accordance with the Declaration of Helsinki.

### Questionnaire design

The questionnaire included the following sections: (1) general background information of the respondent; (2) Tumour therapies and POI; (3) Surgeries or procedures and POI; and (4) ISs and POI. There were 16 questions in total, and full questionnaire details are provided in the Supplementary material.

### Statistical analysis

Data analysis was performed using SPSS (ver. 25.0 IBM, Armonk, NY, USA). Categorical variables are presented as a number (frequency) and percentage. The association between two categorical variables was tested using the chi-square test. Due to the significant variation in the sample size among subgroups, subgroup analyses were not performed based on physicians’ gender, speciality, or hospital type. An overlap existed among respondents regarding age and length of service, with the latter being more representative of work experience. Thus, subgroup analyses were conducted based on physicians’ length of service and hospital type (community, secondary, and tertiary). Values of *p* < 0.05 were considered statistically significant. To better understand the discriminative variables, a correspondence analysis was conducted to visualise the correlation among variables.

## Supplementary Information

**Additional file 1.** Questionnaire.

**Additional file 2 **: **Supplementary Table 1.** Comparison of views on iatrogenic condition(s) to induce POI among respondents with different length of service (years).

**Additional file 3 **: **Supplementary Table 2.** Comparison of views on iatrogenic condition(s) to induce POI among respondents from different levels of hospital.

## Data Availability

Data and materials are summarised in the manuscript, figures, and tables.

## Abbreviations

BS: Bilateral salpingectomy
BTL: Bilateral tubal ligation
CH: Community hospital
CT: Chemotherapy
GnRH-a: Gonadotropin-releasing hormone agonist
H&BS: Hysterectomy with bilateral salpingectomy
ISs: Immunosuppressants
OC: Ovarian cystectomy
PGC: Primordial germ cells
POI: Premature ovarian insufficiency
RT: Radiotherapy
SLE: Systemic lupus erythematosus
TIT: Tumour immunotherapy
TTT: Tumour-targeting therapy
UAE: Uterine artery embolisation
