# Supplementary material for: Knowledge of iatrogenic premature ovarian insufficiency among Chinese obstetricians and gynaecologists: a national questionnaire survey
Source: J Ovarian Res. 2020 Nov 18;13:134. doi: 10.1186/s13048-020-00739-z (PMC7677772; doi:10.1186/s13048-020-00739-z)
Supplement: Supplementary file 1 — Additional file 1. Questionnaire. [file 13048_2020_739_MOESM1_ESM.pdf]

QUESTIONNAIRE

Part 1.General background information.

Q.1 Gender

a) female b) male

Q.2 Age (in years)

a) 18~25 b) 26~35 c) 36~45 d) 46~55 e) >55

Q.3 Years of working

a) ≤5 b) 6~10 c) 11~20 d) >20

Q.4 The level of hospital you are working in.

a) Tertiary hospital b) Secondary hospital

c) Community hospital d) Others___________

Q.4 The type of hospital you are working in.

a) General hospital b) Maternity and children hospital or reproductive hospital

c) Tumour hospital d) Others___________

Q.5 The type of specialty you are committed to.

a) Gynaecologic endocrinologist or reproductive physician b) Gynaecologist

c) Obstetrician-gynaecologist d) Obstetrician e) Gynaecologic oncologist

Part 2.Do you think the following tumour treatments can cause premature ovarian insufficiency?

Q.6 Chemotherapy a) Yes b) No

Q.7 Radiotherapy a) Yes b) No

Q.8 Tumour immunotherapy a) Yes b) No

Q.9 Tumour-targeting therapy a) Yes b) No

Part 3. Do you think the following surgeries or procedures can cause premature ovarian insufficiency?

Q.10 Ovarian cystectomy a) Yes b) No

Q.11 Hysterectomy with bilateral salpingectomy a) Yes b) No

Q.12 Bilateral salpingectomy a) Yes b) No

Q.13 Bilateral tubal ligation a) Yes b) No

Q.15 Uterine artery embolisation a) Yes b) No

Part 4. Immunosuppressants.

Q.16 Do you think immunosuppressants can cause premature ovarian insufficiency?

a) Yes b) No
