# Supplementary material for: Knowledge of iatrogenic premature ovarian insufficiency among Chinese obstetricians and gynaecologists: a national questionnaire survey
Source: J Ovarian Res. 2020 Nov 18;13:134. doi: 10.1186/s13048-020-00739-z (PMC7677772; doi:10.1186/s13048-020-00739-z)
Supplement: Supplementary file 2 — Additional file 2 : Supplementary Table 1. Comparison of views on iatrogenic condition(s) to induce POI among respondents with different length of service (years). [file 13048_2020_739_MOESM2_ESM.docx]

Supplementary Table 1. Comparison of views on iatrogenic condition(s) to induce POI among respondents with different length of service (years).

| Variable | ≤5 | 6~10 | 11~20 | >20 | χ² | p |
| --- | --- | --- | --- | --- | --- | --- |
| RT | 393 (58.1) | 600 (69.7) | 1122 (74) | 1554 (80.1) | 47.5 | <0.01 |
| CT | 365 (53.9) | 510 (59.2) | 1007 (66.4) | 1320 (68) |  |  |
| TIT | 194 (28.7) | 263 (30.5) | 482 (31.8) | 651 (33.6) |  |  |
| TTT | 131 (19.4) | 192 (22.3) | 321 (21.2) | 465 (24) |  |  |
| OC | 382 (56.4) | 466 (54.1) | 857 (56.5) | 1011 (52.1) |  |  |
| H&BS | 221 (32.6) | 294 (34.1) | 600 (39.6) | 865 (44.6) |  |  |
| UAE | 194 (28.7) | 268 (31.1) | 520 (34.3) | 693 (35.7) |  |  |
| BS | 130 (19.2) | 200 (23.2) | 379 (25) | 537 (27.7) |  |  |
| BTL | 98 (14.5) | 132 (15.3) | 246 (16.2) | 303 (15.6) |  |  |
| ISs | 196 (29) | 255 (29.6) | 478 (31.5) | 594 (30.6) |  |  |

Abbreviations: RT: radiotherapy; CT: chemotherapy; TIT: tumour immunotherapy; TTT: tumour-targeting therapy; OC: ovarian cystectomy; H&BS: hysterectomy with bilateral salpingectomy; UAE: uterine artery embolisation; BS: bilateral salpingectomy; BTL: bilateral tubal ligation; ISs: immunosuppressants.
