# Supplementary material for: Knowledge of iatrogenic premature ovarian insufficiency among Chinese obstetricians and gynaecologists: a national questionnaire survey
Source: J Ovarian Res. 2020 Nov 18;13:134. doi: 10.1186/s13048-020-00739-z (PMC7677772; doi:10.1186/s13048-020-00739-z)
Supplement: Supplementary file 3 — Additional file 3 : Supplementary Table 2. Comparison of views on iatrogenic condition(s) to induce POI among respondents from different levels of hospital. [file 13048_2020_739_MOESM3_ESM.docx]

Supplementary Table 2. Comparison of views on iatrogenic condition(s) to induce POI among respondents from from different levels of hospital.

| Variable | Community or others | Secondary | Tertiary | χ² | p |
| --- | --- | --- | --- | --- | --- |
| RT | 502 (112.1) | 1779 (72.8) | 1388 (79.5) | 300.8 | <0.01 |
| CT | 477 (106.5) | 1565 (64) | 1160 (66.5) |  |  |
| TIT | 299 (66.7) | 852 (34.8) | 439 (25.2) |  |  |
| TTT | 224 (50) | 595 (24.3) | 290 (16.6) |  |  |
| OC | 370 (82.6) | 1256 (51.4) | 1090 (62.5) |  |  |
| H&BS | 260 (58) | 948 (38.8) | 772 (44.2) |  |  |
| UAE | 158 (35.3) | 724 (29.6) | 793 (45.4) |  |  |
| BS | 174 (38.8) | 585 (23.9) | 487 (27.9) |  |  |
| BTL | 108 (24.1) | 394 (16.1) | 277 (15.9) |  |  |
| ISs | 280 (62.5) | 817 (33.4) | 426 (24.4) |  |  |

Abbreviations: RT: radiotherapy; CT: chemotherapy; TIT: tumour immunotherapy; TTT: tumour-targeting therapy; OC: ovarian cystectomy; H&BS: hysterectomy with bilateral salpingectomy; UAE: uterine artery embolisation; BS: bilateral salpingectomy; BTL: bilateral tubal ligation; ISs: immunosuppressants.
